# The complete chloroplast genome of monocot plant, *Maianthemum dilatatum*

**DOI:** 10.1080/23802359.2018.1524723

**Published:** 2018-10-25

**Authors:** Raman Gurusamy, Eun Mi Lee, Gi Heum Nam, Byoungyoon Lee, SeonJoo Park

**Affiliations:** a Department of Life Sciences, Yeungnam University, Gyeongsan, Gyeongsan-buk, Republic of Korea;; b Plant Resources Division, National Institute of Biological Resources of Korea, Incheon, Republic of Korea

**Keywords:** *Maianthemum dilatatum*, chloroplast genome, genome evolution, Nolinoideae

## Abstract

The complete chloroplast genome sequence of *Maianthemum dilatatum* is sequenced and analyzed. The chloroplast genome is 156,921 bp, with 36.7% GC content. A pair of inverted repeats of 26,468 bp is separated by a large single-copy region (85,554 bp) and a small single-copy region (18,431 bp). It encodes 86 protein-coding genes, 38 tRNA genes and 8 rRNA genes. Of 132 individual genes, 19 genes are duplicated in the IR regions, while 14 genes are encoded with one intron and three genes with two introns.

The plant organelle, chloroplast (cp) has evolved from an ancient endosymbiotic bacterium, cyanobacteria (Gray 1999) that involves photosynthesis and provides energy for green plants and algae (Douglas 1998) and encodes its own genomic DNA which is highly conserved it their plants. The cp genome usually a typical circular moleculeencodes two inverted repeats separated by large single copy (LSC) and small single copy (SSC) regions (Jansen et al. 2005). When compared with mitochondrial and nuclear genomes, chloroplast genomes are more conserved in term of its gene content, organization and structure, and it has been usually applied for understanding the genome evolution and genetic diversity (Raubeson and Jansen 2005).

The rhizomatous perennial monocot flowering plant, *Maianthemum dilatatum* (Alph.Wood) A. Nelson and J.F. Macbr belong to Nolinoideae subfamily. The mashed leaves have been applied to boils, burns, cuts, and wounds, fruit for the treatment of tuberculosis, root for the treatment of reverse sterility and sore eyes (Pojar and Mackinnon 2005). Due to its medicinal property, it is very important to understand the genetic structure of this species. So, in the present study, the plant, *M. dilatatum* was collected from the Dok-do Island of Korea and the chloroplast genome of *M. dilatatum* is sequenced, assembled, and characterized. The voucher was deposited at the herbarium of Yeungnam University (YU), Gyeongsan, South Korea.

The chloroplast genome is a double-stranded, circular DNA with 156,921 bp in length and GC content is 36.7%. The genome sequence was deposited in GenBank (GenBank accession number: MF150041). The chloroplast genome of *M. dilatatum* exhibits a typical quadripartite structure. The large and single-copy (LSC and SSC) region of *M. dilatatum* are 85,554 bp, 18,431 bp, respectively, and two parts of an inverted repeat (IR) of 26,468 bp each (Figure 1). There are a total of 132 genes predicted in the genome, of which 113 unique, including 79 protein-coding genes, 30 tRNA genes, and 4 rRNA genes. A total of 19 genes duplicated in the IR regions such as 7 protein-coding genes, 8 tRNAs, and 4 rRNA genes. Twelve protein-coding genes and 5 tRNAs have introns. The newly characterized chloroplast genome sequence of *M. dilatatum* will provide an essential information for further studies to understand the phylogenetic relationships and biodiversity of the Nolinoideae subfamily in monocots.

**Figure 1.**
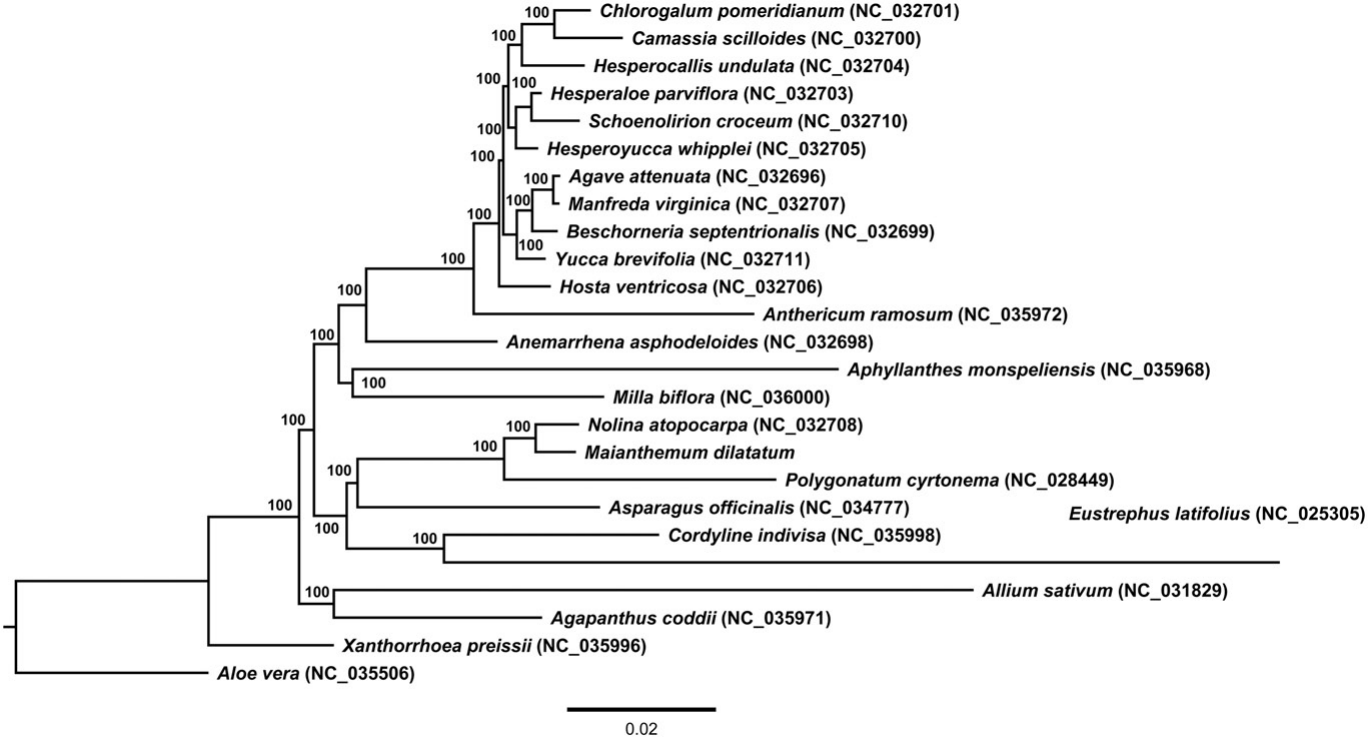
Phylogenetic tree of *Maianthemum dilatatum* and related taxa using the complete chloroplast genome sequences. The tree was constructed by maximum likelihood (ML) analysis using the RaxML program and the GTR + I nucleotide model. The stability of each tree node was tested by bootstrap analysis with 1000 replicates. Bootstrap values are indicated on the branches, and the branch length reflects the estimated number of substitutions per 1000 sites.
